# Membrane Carriers and Transporters in Kidney Physiology and Disease

**DOI:** 10.3390/biomedicines9040426

**Published:** 2021-04-14

**Authors:** Marek Drozdzik, Maria Drozdzik, Stefan Oswald

**Affiliations:** 1 Department of Experimental and Clinical Pharmacology, Pomeranian Medical University, 70-111 Szczecin, Poland; 2 Faculty of Medicine, Medical University of Lodz, 90-419 Lodz, Poland; mariadrozdzik@gmail.com; 3 Institute of Pharmacology and Toxicology, Rostock University Medical Center, 18051 Rostock, Germany; stefan.oswald@med.uni-rostock.de

**Keywords:** drug transporters, kidney, kidney pathology

## Abstract

The growing information suggests that chronic kidney disease may affect expression and function of membrane carriers and transporters in the kidney. The dysfunction of carriers and transporters entails deficient elimination of uremic solutes as well as xenobiotics (drugs and toxins) with subsequent clinical consequences. The renal carriers and transporters are also targets of drugs used in clinical practice, and intentional drug–drug interactions in the kidney are produced to increase therapeutic efficacy. The understanding of membrane carriers and transporters function in chronic kidney disease is important not only to better characterize drug pharmacokinetics, drug actions in the kidney, or drug–drug interactions but also to define the organ pathophysiology.

## 1. Introduction

**Figure 1:**
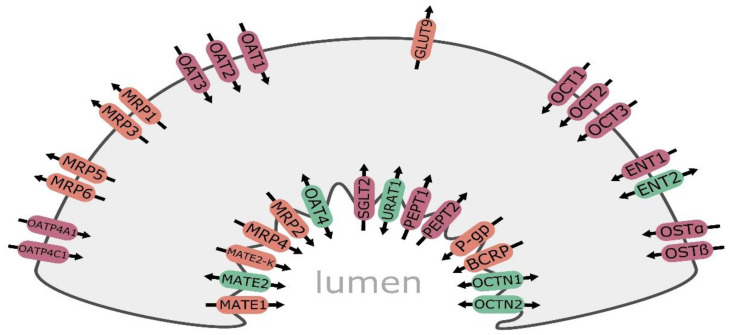
Major drug transporters in the kidney renal epithelial cell: ENT1—equilibrative nucleoside transporter 1; ENT2—equilibrative nucleoside transporter 2; GLUT9—facilitative glucose transporter 9; MATE1—multidrug and toxin extrusion protein 1; MATE2—multidrug and toxin extrusion protein 2; MATE2-K—multidrug and toxin extrusion protein 2 kidney-specific; MRP1—multidrug resistance-associated protein 1; MRP2—multidrug resistance-associated protein 2; MRP3—multidrug resistance-associated protein 3; MRP4—multidrug resistance-associated protein 4; MRP5—multidrug resistance-associated protein 5; MRP6—multidrug resistance-associated protein 6; P-gp—multidrug resistance protein 1/P-glycoprotein; OAT1—organic anion transporter 1; OAT2—organic anion transporter 2; OAT3—organic anion transporter 3; OAT4—organic anion transporter 4; OATP4A1—organic anion transporting polypeptide 4A1; OATP4C1—organic anion transporting polypeptide 4C1; OCT1—organic cation transporter 1; OCT2—organic cation transporter 2; OCT3—organic cation transporter 3; OCTN1—organic cation/carnitine transporter 1; OCTN2—organic cation/carnitine transporter 2; OSTα—organic solute transporter α; OSTβ—organic solute transporter β; PEPT1—peptide transporter 1; PEPT2—peptide transporter 2; SGLT2—sodium-glucose co-transporter 2; URAT1—urate transporter 1. Efflux transporters/carriers are highlighted in orange, influx carriers in red, and bidirectional carriers in green.

Kidney plays a key role in elimination of hydrophilic molecules of both endogenous and exogenous origin. In the organ, both glomerular filtration as well as tubular secretion and reabsorption are principal components determining renal clearance. The carriers and transporters are expressed on basolateral and apical membranes of proximal, distal, and collecting tubule epithelia providing bidirectional movement of substrate molecules and are subdivided into two major superfamilies, i.e., ATP-binding cassette transporters (ABC, consisting of about 50 members, subdivided into 7 families) and solute carriers (SLC, more than 400 membrane proteins grouped into over 60 families). In kidney tubule cells, members of the ABC-superfamily transporters are expressed and provide efflux functions, i.e., multidrug resistance protein 1/P-glycoprotein (MDR1, P-gp and *ABCB1*), multidrug resistance-associated protein 2 (MRP2, *ABCC2*), 3 (MRP3, *ABCC3*), and 4 (MRP4, *ABCC4*). The SLC carriers are engaged in cellular influx and/or cellular efflux of molecules. In the kidney, the following SLC carriers can be found: organic anion transporting polypeptide 4C1 (OATP4C1, *SLCO4C1*), organic anion transporter 1 (OAT1, *SLC22A6*), 2 (OAT2, *SLC22A7*), 3 (OAT3, *SLC22A8*), 4 (OAT4, *SLC22A11*), and organic cation transporter 2 (OCT2, *SLC22A2*) as well as organic cation/carnitine transporter family members OCTN1 (*SLC22A4*) and OCTN2 (*SLC22A5*), multidrug and toxin extrusion protein family (MATE1/*SLC47A1,* MATE2/*SLC47A2,* and MATE2-K/*SLC47A2*), peptide transporters 1 and 2 (PEPT1/*SLC15A1* and PEPT2/*SLC15A2*), equilibrative nucleoside transporters 1 (ENT1/*SLC29A1*) and 2 (ENT2/*SLC29A2*), and then urate transporter 1 (URAT1, *SLC22A12*) and sodium-glucose co-transporter 2 (SGLT2, *SLC5A2*) (Figure 1). Membrane carriers/transporters being members of the SLC families (OCTs, OATs, and MATEs) are more abundantly expressed than ABC-family transporters. The list of the most abundant renal SLC carriers include OAT1, OAT3, OCT2, and MATE1, while P-gp, MRP2, and MRP4 belong to the predominant ABC transporters [1,2]. Similar to other segmental organs (e.g., gastrointestinal tract), it seems that distribution of transporters along nephron may be segment specific. Proximal, distal, and collecting duct cells can be characterized by a different panel of membrane transporters, most of which were characterized in the proximal tubule cells. However, OCT2, GLUT9, and SGLT2 are examples of carriers exclusively expressed in the kidney proximal tubule cells, whereas MRP3 transporter confines to the distal tubule cells [3,4].

The transporters, both in the kidney and other complex organs, function in coordinated mode, which allows transmembrane shift of cation, anion, or zwitterion substrates. The SLC carriers, taking anions (OATs) or cations and zwitterions (OCTs, OCTNs, MATEs), can provide both uptake (mostly) and efflux functions. In a facilitative mode, the SLC carriers shift substrates along electrochemical gradients, which does not require energy input. In less frequent, active mode, SLC transporters provide transport activity against gradient of a substrate by coupling it to electrochemical gradient of a co-transported ion (e.g., Na^+^ and H^+^) or solute. The ABC transporters, taking anions (MRPs) or cations (P-gp) operate primarily as active transporters, and shuttle substrates against their electrochemical gradients, for which energy from ATP hydrolysis is used [4].

The importance of knowledge on kidney drug transporter engagement in drug handling is expressed in the Food and Drug Administration (FDA) and the International Transporter Consortium recommendations. P-gp, OAT1, OAT3, OCT2, MATE1, and MATE2-K are listed for evaluation in drug–drug interaction studies [5,6]. Those transporters participate in endogenous compounds (creatinine and uremic toxins) and drug handling within the kidney with resultant endogenous compound–drug–drug interactions. Table 1 shows the list of substrates of the major kidney membrane carriers and transporters.

## 2. Function of Drug Transporters and Carriers

Membrane transporters localized in both basolateral and apical membranes of kidney tubule cells, which function in coordinated manner, provide a mechanism of vectorial transport across cell membranes in both directions, serving as key mediators of elimination and absorption pathways. Dysfunction and/or inhibition of organic anion (e.g., OATs, OATPs, and MRPs) or cation (OCTs, P-gp, and MATEs) uptake carriers and/or efflux transporters may lead to reduced drug elimination (in the case of both uptake and efflux transport), increased tubule cell drug accumulation (dysfunction of efflux transporters), or drug competition at carrier or transporter site. Those effects may mediate altered drug pharmacokinetics, drug–drug interactions, or drug toxicity (drug substrates of selected kidney carriers and transporters are presented in Table 1). The function and role of several transporters was defined, and the clinical relevance of some of the kidney-expressed transporters and carriers are highlighted by regulatory agencies (P-gp, OAT1, OAT3, OCT2, MATE1, and MATE2-K) [5,6]. Several of the potential interactions were documented in clinical settings [7,8,9,10,11].

Organic cations (OCs) are handled by OCTs (as uptake system) and P-gp and MATEs (as efflux transporters). The function of MATE1 transporter is pH-dependent (at and above pH 7.4, it functions as an uptake carrier, whereas it provides efflux activity at pHs of less than 7.4), and in the kidney proximal tubule in vivo (pH < 7.4), it functions as an efflux transporter, which is rather unusual for an SLC drug carrier. Cellular uptake of OCs in the basolateral membrane of the kidney tubular cell is mediated mainly by OCT2, which utilizes negative potential difference within the cell produced by the basolateral Na^+^-K^+^-ATPase. On the apical membrane, cations can be shifted by ATP-dependent transporters, mainly P-gp into nephron lumen. MATE1 (SLC carrier), a cation export transporter, which shuttles OCs in electroneutral exchange with proton (H^+^) owing the electrochemical gradient favoring movement of H^+^ into the cells, also operates in the apical membrane [12]. Function of breast cancer resistance protein (BCRP/*ABCG2*) in human kidney is a bit controversial. Proteomic studies evidenced that BCRP protein levels were below the lower limit of quantification [1,2]. Contrary, immunohistochemical analysis revealed the proximal tubule brush border membrane localization of BCRP in human kidneys (immunohistochemistry is a more sensitive, but less specific method in comparison to targeted proteomics). The function of BCRP in the kidney was positively verified by fumitremorgin C and nelfinavir (BCRP inhibitors)-mediated inhibition of Hoechst 33,342 dye efflux from primary human proximal tubule cells [13]. Those findings suggest that BCRP may contribute (if any) to organic cation excretion in the kidney.

The transport mechanisms of OCTN1 and OCTN2 are substrate dependent and quite different from each other. At the apical membrane, OCTN1 mediates Na^+^-dependent reabsorption (e.g., ergothioneine) from the filtrate or contributes to tubular efflux secretion driven by the acidic pH in the lumen (cations). OCTN2 functions as a Na^+^-dependent co-transporter (e.g., L-carnitine), but can also provide Na^+^-independent activity. OCTN2 (like OCTN1) may participate in renal reabsorption of zwitterions (e.g., L-carnitine) or secretion of xenobiotic OC depending on its mode of transport [14,15].

In the kidney, organic anions (OAs) are transported by MRPs, OATs, OATPs, and of those are bidirectionally shuttled by OAT4 as well as URAT1 (to date, 10 OAT isoforms have been identified in human, but not all are functionally characterized). The major carriers engaged in uptake functions, OAT1 and OAT3, are driven by Na^+^ active outward transport generated by the basolateral Na^+^/K^+^-ATPase activity. The Na^+^/K^+^-ATPase creates Na^+^ gradient, which facilitates sodium dicarboxylate cotransporters to move Na^+^ and dicarboxylate (e.g., α-ketoglutarate for OAT1 and OAT3, succinate for OAT2) into the cell. Consequently, high intracellular concentration of dicarboxylates promotes OAs uptake across the basolateral membrane in exchange for dicarboxylates. Human OAT2 is mainly expressed in the liver, but was also retrieved in the kidney [16]. The apical OATs, i.e., OAT4 and URAT1 demonstrate multiple biological activities. The URAT1 exchanges extracellular urate with intracellular organic anions (lactate and nicotinate), which leads to urate reabsorption from the renal tubule lumen [17]. OAT4 can function both as an influx and efflux carrier. It can reabsorb estrone sulfate and urate through OA/dicarboxylate or OA/OH^-^ exchange mode. As an influx transporter, it can release p-aminohippurate (PAH) into the tubule lumen via PAH/Cl^-^ exchange [18].

In the kidney, OATPs are represented mainly by OATP4C1, which is expressed in the basolateral membrane of proximal tubule cells of the S2 segment (as demonstrated in transgenic rats expressing human OATP4C1). The exact mechanisms of carrier functions are unclear, but the transport by OATPs is Na^+^-independent and is assumed to act on OA/OA (bicarbonate, glutathione (GSH), and GSH conjugates) exchanger mode, coupling cellular uptake of organic compounds with efflux of intracellular OAs [19].

Human basolateral membrane of renal proximal tubular cells is endowed with organic solute transporters (OST)-α and -β. The operative mode of these carriers is not entirely defined, but its facilitated diffusion transport mode was demonstrated to be unaffected by depletion of intracellular adenosine triphosphate or by changes in transmembrane Na^+^, K^+^, H^+^, or Cl^−^ concentration gradients. OSTs function in coordinated fashion with apical sodium-dependent bile acid uptake transporter (ASBT), which shuttle bile acids and sterols, thus participating in their reabsorption [20].

The apical efflux of organic anions in the proximal tubules is mediated by the energy-dependent ABC transporters MRP2 and MRP4. These transporters, using energy generated from ATP hydrolysis, actively transport their substrates out of cells against their concentration gradients. MRP2 and MRP4 (apical) demonstrate substrate overlap with OAT1 and OAT3 (basolateral) and may provide coordinated renal excretion of certain anionic molecules or drugs. MRP3 is localized in the distal tubule basolateral membrane, where the transporter carries glucuroconjugated compounds and other molecules from the internal tubular cell into the blood, and may be engaged in reabsorption of conjugated steroids [16,21].

The function of drug transporters in kidney tubule cells is well defined, especially for proximal tubules, but there is only emerging evidence on their function in glomeruli. In-depth proteomic profiling identified that transporters and carriers constituted 2% of all identified proteins of the glomerulus of normal human kidney, among them are SLCO4A1, monocarboxylate transporter 7 (*SLC16A6*), and multidrug resistance protein 3 (*ABCB4*) [22]. However, the functional role of these transporters in the glomerulus was not defined. mRNA expression coding for OATP1A2, OATP2B1, OATP4A1, OAT3, and PEPT1 was found in the human immortalized podocyte cell line (obtained by infection of primary cultures with a hybrid Adeno5/SV40). OATPs function was documented in penicillin G uptake study. Efflux transporters in podocytes are represented by P-gp (mRNA expression, immunofluorescence, and function assessed by rhodamine-123 transport) [23,24]. In another study, an immunostaining revealed plasma membrane amino-acid transporter (PMAT, *SLC29A4*) in human podocytes (with minimal expression in tubular cells), which may be related to monoamine signaling pathways in the kidney. A functional experiment defined engagement of PMAT in puromycin aminonucleoside (PAN) (a classic podocyte toxin that induces massive proteinuria and severe glomerulopathy) transport. These findings suggest that PMAT specifically expressed in podocytes may play an important role in PAN-induced kidney injury [25]. Podocytes were also found to express several GLUTs, and of these, GLUT1 and GLUT4 were shown to be insulin responsive, and their dysfunction was proposed to be associated with development of diabetic nephropathy [26].

## 3. Effects on Endogenous Substrates

Drug transporters and carriers in the kidneys not only are implicated in drugs and other xenobiotics handling but also serve in endogenous substrates transport. Those transporters and carriers participate in elimination of creatinine, uric acid, or several uremic toxins (which in clinical settings can also enter into interactions with drugs).

Creatinine is transported by OCT2 and MATEs. Contribution of transporters to creatinine elimination underlies the observation that creatinine clearance usually exceeds the glomerular filtration rate (GFR), the phenomenon more prominent in deteriorated kidney function states (especially in patients with glomerular disorders) [27]. Data based on iothalamate and iohexol as alternative GFR markers suggest up to 24% and 38% contribution of active secretion to creatinine clearance, respectively [28]. The molecule is taken up by OCT2 carrier in the basolateral membrane, and then is shuttled and eliminated by MATEs localized in the apical site of the tubule cells. In support, the genome-wide association studies showed a link between the *SLC22A2* gene (coding OCT2) and both serum creatinine and estimated GFR [29]. There is also evidence for OAT2 participation, and involvement of OAT4 and OCT3 has been proposed [30]. Competition for the transporters and/or inhibition may lead in clinical settings to reduced creatinine elimination and increased creatinine concentrations. This effect was documented for several drugs, i.e., trimethoprim, cimetidine, cobicistat, dolutegravir, pyrimethamine, famotidine, ranolazine, and rilpivirine [30,31,32,33,34].

Uric acid is freely filtered in the glomerulus, and then is mostly reabsorbed by proximal tubular apical urate transporters, with only a small portion secreted back into the filtrate via the distal proximal tubule. The net balance of uric acid in the kidney depends on function of carriers and transporters providing both reabsorptive and excretory functions. The uptake of uric acid from blood into tubular cells is mainly mediated by OAT1 and OAT3, whereas excretion of uric acid from tubular cells to the nephron lumen is governed by reabsorption carriers (and thus determining urate excretion), i.e., URAT1 (major urate reabsorption transporter) and OAT4 as well as efflux transporters (actively secreting uric acid from tubular cells to the lumen) MRP4, sodium-dependent phosphate transport protein, and BCRP [35]. The mutations of the BCRP transporter gene, *ABCG2*, were related with hyperuricemia. A general non-synonymous single nucleotide polymorphism (SNP) in *ABCG2* Q141K had a negative effect on BCRP function [36]. Interactions of both endogenous molecules and drugs with transporters and carriers of uric acid may impact its plasma levels. Increased glucose transport by GLUT9 (high-capacity urate carrier in humans) increases the speed of uric acid reabsorption [37]. Probenecid, a uricosuric agent, targets many kidney transporters, i.e., inhibits OAT1 and OAT3, and thus produces hyperuricemia and drug–drug interactions (leading to increased blood drug concentrations), which is a factor limiting its clinical use. However, reduced tubular uptake of several drugs (cidofovir and tenofovir) is explored in clinical settings to reduce drug-induced kidney injury [38]. Other medications can also regulate uric acid levels by targeting URAT1. Lesinurad, a selective URAT1 and uric acid reabsorption inhibitor, is used to reduce uric acid via increased fractional excretion of uric acid in a dose-dependent manner [39]. Other drugs possess mild-to-moderate URAT1 inhibition activity, such as fenofibrate and losartan, and may be useful to control hyperuricemia and gout in patients with dyslipidemia and hypertension or heart failure, respectively [40,41].

The kidney tubule uptake carriers OAT1 and to lesser extent OAT3 shuttle uremic solutes being small organic anions (p-hydroxyhippuric acid, 3-carboxy-4-methyl-5-propyl-2-furanopropanoic acid (CMPF), hippuric acid, indoleacetic acid, indoxyl sulfate, uric acid, and xanthine) [42]. Similar to other abovementioned carriers of endogenous compounds, these transporters can be considered as potential drug interaction sites. Ketoprofen or diclofenac (non-steroidal anti-inflammatory drugs, NSAIDs), which produce inhibitory activity against OAT1 and OAT3 carriers, can significantly decrease the renal clearance of indoxyl sulfate, leading to its systemic accumulation. Increased exposure to the toxin can in turn contribute to the progression of indoxyl sulfate-induced cardiovascular disease and, in part, explain the pathogenesis of analgesic nephropathy [43,44]. Experimental findings suggest that sartans (losartan and valsartan) and furosemide may compete with uremic toxins for OAT carriers [44]. OATP4C1 facilitates the excretion of asymmetric dimethylarginine (ADMA), guanidine succinate (GSA), and trans-aconitate, and its dysfunction may lead to the toxin accumulation in kidney failure. It was demonstrated that statins (fluvastatin and pravastatin) increased the expression and function of OATP4C1 via up-regulation of *SLCO4C1* promoter activity, resulting in the reduction in uremic toxins and blood pressure [45].

## 4. Effects on Drug Pharmacokinetics and Drug–Drug Interactions (DDIs)

The transporter pathways in the kidney are also engaged in coordinated excretion of drugs. In kidney tubule cells, cationic agents are taken up by the basolateral membrane located OCT2, a predominant member of OCTs in the kidney, but a contribution of OCT3 is also postulated. Transporters operating in the apical membrane, i.e., P-gp, MATEs, and OCTNs, are engaged in cationic drug transfer from the cell into urine. OCT2 was demonstrated to be a carrier especially of OCs with relatively low molecular weight (<500 Da) and small size (<4 Å diameter) [46]. To OCT2 substrates belong, among others, metformin [47], histamine H2 blockers (cimetidine, ranitidine, famotidine) [48], HIV protease inhibitors (lamivudine, zalcitabine) [49], ß-blockers (metoprolol and propranolol) [50], and some platinum compounds (oxaliplatin, cisplatin) [51] (Table 1). The excretory function for cationic drugs is provided by P-gp, MATEs, OCTNs. MATE1, MATE2, and MATE2-K, which efflux type I organic cations of low molecular weight and size, whereas P-gp is engaged in transport of rather bulkier (>500 Da) cationic compounds, and OCTNs play an auxiliary role. MATEs shuttle metformin [47], cimetidine [52], platinum agents (oxaliplatin) [53], or fluoroquinolones (ciprofloxacin, enoxacin, gatifloxacin, levofloxacin, and norfloxacin) [54]. The most important P-gp substrates of clinical relevance are digoxin and other cardiovascular agents [55], immunosuppressants (cyclosporin A, tacrolimus, and sirolimus) [56], antiretrovirals (ritonavir and saquinavir) [57], phenobarbital, phenytoin [58], anticancer (paclitaxel and vinblastine) [55], or statins (atorvastatin) [59] (Table 1).

Vectorial drug transport was nicely documented for metformin, which is a substrate of basolateral uptake carrier OCT2, and subsequently is excreted into the urine by apical MATE1 and MATE2-K (MATE2-K is a splice variant MATE2, which functions as an efflux transporter exclusively expressed in the apical membrane of kidney proximal tubular cells) [47]. The same transporters participate in urine elimination of oxaliplatin [53]. Difference between cisplatin and oxaliplatin in MATE2-K affinity may explain high nephrotoxic potential of cisplatin. Namely, MATE2-K shuttles oxaliplatin with higher affinity than cisplatin, which results in higher intracellular accumulation of cisplatin promoting its nephrotoxic activity [51].

Coordinated function of kidney transporters engaged in anion handling is reflected in renal methotrexate (MTX) excretion. The drug is taken up by basolateral carriers OAT1 and OAT3 and, afterwards, is eliminated into urine via apical MRP2 and MRP4 transporters. A role of OAT4C1, a basolateral uptake carrier, in the MTX elimination in the kidney is also postulated [60,61]. OAT and MRP carriers and transporters operating in concert participate in kidney elimination of numerous clinically important drugs (Table 1), among them are diuretics (thiazides, furosemide) [62,63], NSAIDs (ibuprofen, indomethacin, ketoprofen, or salicylate, but not acetylsalicylate) [64], angiotensin II antagonists (losartan) [65], β-lactam antibiotics (cephalothin, cefoperazone, cefazolin, ceftriaxone, cephaloridine, cefotaxime, cefadroxil, cefamandole, and penicillin G) [66], and antiviral drugs (adefovir, cidofovir, and tenofovir) [67]. OAT1 and OAT3 have largely overlapping substrate specificity, but OAT3 prefers more bulky and lipophilic organic anionic substrates, e.g., glucuronide conjugates [68].

The kidney drug transporter function (produced by inhibitors or inducers) or competition for a transporter/carrier (drug–drug or drug–endogenous compound interactions) can affect kidney drug elimination and thus drug pharmacological actions in a significant manner. There are several clinically important interactions reported in literature. NSAIDs, especially ibuprofen, naproxen, are known inhibitors of OATs, and thus can impact tubular uptake of OAT substrates. MTX is taken up from the blood via OAT carriers in the kidney, and NSAIDs-dependent inhibition of OAT carriers produce impaired renal elimination of MTX, which leads to the drug accumulation and its toxicity manifesting mostly as severe bone marrow suppression [7]. Interaction of MTX with co-trimoxazole (in fact sulfamethoxazole, but not trimethoprim, share the same kidney elimination pathway mediated by OATs) have also been documented. The competition for tubular secretion mechanisms results in significant increase in systemic free MTX levels and higher rate of side effects (mainly myelotoxicity) [8]. As MTX or NSAIDs are also recognized by MRP2 and MRP4, the interaction at efflux carriers that leads to decreased urinary excretion could contribute to the observed changes of the systemic drug exposure.

Similar to anionic drug transport system interactions, drug transporters for cationic drug moieties can also constitute a site for drug–drug competition. DDIs at the level of P-glycoprotein in the proximal tubule and elsewhere are thought to explain the known digoxin–quinidine or digoxin–verapamil interactions resulting in increased systemic digoxin concentrations and then toxicity, including arrhythmias [9]. Competition at OCT2 uptake carrier is another site of potential DDIs. Dolutegravir, an OCT2 inhibitor, was evidenced to significantly increase metformin plasma exposure, which can be partially explained by OCT2 inhibition. To balance increased metformin systemic exposure, dose adjustments of metformin should be considered to maintain optimal glycemic control when patients are starting/stopping dolutegravir while taking metformin [10]. MATEs cooperating with OCTs in the kidney are also sites for DDIs. Cisplatin, as mentioned above, is shuttled by MATE1 located in the basolateral membrane. Inhibition of MATE1 and MATE2-K by ondansetron resulting in increased intracellular concentrations of cisplatin can potentiate its nephrotoxicity [11].

## 5. Transporters as Therapeutic Drug Targets

Drug transporters and carriers in the kidney are also directly targeted to produce therapeutic effects or intentional drug–drug interactions in order to potentiate drug responses. Kidney membrane transporters can be responsible for DDIs of drugs being their inhibitors, inducers, or substrate competitor at transporter levels. Some of potential interactions were described, but clinical significance was not always defined. However, when using in clinical setting drugs targeting specific transporter system (see Table 1) a possibility of interaction should be considered, especially in patients with poor renal function, which can further complicate the clinical picture [69,70,71,72,73,74,75,76].

Transporters engaged in uric acid excretion are also therapeutic targets. Lesinurad is a drug providing uricosuric activity via direct inhibition of proximal tubule URAT1 (*SLC22A12*) carrier as well as another proximal tubule uric acid reabsorption transporter, i.e., OAT4 (*SLC22A11*). Reduced reabsorption rate of uric acid in the apical membrane entails downregulation of its systemic concentrations. From functional point of view, it should be also stated that lesinurad exchanges lactate (predominantly) for uric acid, and under the treatment, an increased serum lactate concentration should be taken into account. The approved indication for lesinurad is treatment of gout in combination with a xanthine oxidase inhibitor (XO), e.g., allopurinol, febuxostat, in adult patients who have not achieved target serum uric acid levels with a XO inhibitor alone [77]. Probenecid can also be considered as an agent, which reduces uptake of uric acid in the proximal tubule cell, thus providing uricosuric action. The drug mode of action relies on its inhibitory actions on OAT1 and OAT3 [78].

Some drugs, with principal mode of action other than kidney transporter function modulation, were defined as inhibitors of URAT1 carrier and in clinical situations with co-existing hyperuricemia can be of preference. Losartan, an angiotensin AT1 receptor blocker and fenofibrate, hypolipemic drug of complex mode of actions (mainly activation of lipoprotein lipase) are also URAT1 inhibitors. Therefore, losartan can be the preferred option of treatment of patients with elevated uric acid levels/gout and arterial hypertension [79], whereas fenofibrate in patients with dyslipidemia and hyperuricemia [80].

Drugs targeting SGLT2 (*SLC5A2*), i.e., gliflozins (canagliflozin, empagliflozin, and dapagliflozin), are used in the treatment of diabetes mellitus. The agents inhibit function of SGLT2 carrier in the proximal tubules, which prevents the reabsorption of glucose (and indirectly natriuresis along with this process). The drugs do not only offer better glycemic control but also seem to reduce hospitalization for heart failure and progression of renal disease regardless of existing atherosclerotic cardiovascular disease or history of heart failure [71].

Some intentional DDIs at the transporters/carriers’ level in the kidney are used in clinical practice. Probenecid is a multiple OATs inhibitor, and this activity is explored to inhibit drug excretion and elimination (and thus increasing their systemic concentrations) or reducing uptake of nephrotoxic agents by tubule cells (and decrease their toxicity). Probenecid is recommended in co-administration with antimicrobial agents, which are substrates of OATs (mainly OAT1 and OAT3), in order to increase their systemic concentrations (and also to prolong dosing interval) [81]. Competition of probenecid with cephalosporines (cefuroxime, cephalexin, cefazolin, cefuroxime, cefaclor, cefotaxime, and ceftazidime) and penicillins (ampicillin, amoxicillin, and flucloxacillin) for OATs reduces the rate of renal tubular secretion/elimination. This type of interaction aims at an increase in therapeutic efficacy of antimicrobials, as a 2-fold to 4-fold drug concentration elevation has been demonstrated for various penicillins and cephalosporins. The main indication for the combined use is treatment of uncomplicated gonorrhea, syphilis, and cellulitis [82,83].

Cidofovir, an antiviral drug, is eliminated mostly as a parent compound through renal excretion, and its nephrotoxicity, due to excessive drug accumulation in renal proximal tubule cells, is the dose-limiting factor. The drug is an OAT1 substrate, and co-administration of probenecid via inhibition of the OAT-mediated uptake markedly reduces nephrotoxic potential of the drug and supports the use of probenecid as nephroprotectant during cidofovir therapy [84]. Nowadays, co-administration of probenecid with cidofovir is required by FDA to protect patients against cidofovir-induced nephrotoxicity [4].

Probenecid via inhibitory actions on kidney tubule cell uptake carriers OAT1 and OAT3, which warrant its application in clinical medicine, is also used to reduce urinary excretion of some doping agents, not allowed to be used by sport professionals. This strategy has been explored to mask the use of banned performance-enhancing drugs, e.g., anabolic-androgenic steroids [85].

## 6. Effects of Kidney Failure on Renal Drug Transporters

The expression and function of drug carriers and transporters in the kidney is best described in healthy state, but it is obvious that the organ pathology involves dysregulation of membrane carriers and transporters. However, most of the available information is derived from experimental studies. Human evidence is very scarce, and stem from in vitro observations and few clinical findings. Those studies suggest that kidney uptake carrier system consisting of OATs and OCTs is more vulnerable than export transporters. This phenomenon is observed in immortalized renal cells cultures, where a lot of cell models demonstrate deficiency of uptake functions (and it constitutes a substantial drawback of renal cell lines applications) [86,87]. Exposure of human kidney-2 cells (HK-2, a proximal tubule cell model) to sera obtained from rats with chronic renal failure (CRF) resulted in a significant downregulation of the protein expression levels of Oat3, organic anion-transporting polypeptide 1 (Oatp1), and P-gp, whereas levels of Mrp2, Mrp4, and Oatp2 were significantly upregulated [88].

Findings in clinical kidney samples also support evidence of predominant deficit in uptake carriers. Significant reduction in *SLC22A6* (OAT1) mRNA expression was defined in renal biopsy specimens from patients with different types of nephropathy, i.e., lupus nephritis, IgA nephropathy, focal glomerular sclerosis, membranoproliferative glomerulonephritis, membranous glomerulonephropathy, mesangial proliferative glomerulonephropathy, in comparison to the normal controls. The expression levels of *SLC22A8*, *SLC22A7*, and *SLC22A11* were not significantly altered in comparison to the normal kidneys. The same study also provided functional verification of the changes in OATs and showed significant correlation between cefazolin (OATs substrate) elimination rate constant and the values of phenolsulfonphthalein test and mRNA levels of *SLC22A8* [89].

Another observation supports findings of decreased OATs function in severe renal dysfunction patients (creatinine clearance (CrCl) < 30 mL/min estimated by the Cockcroft-Gault formula). It was evidenced that kidney failure was associated with plasma accumulation and reduced renal clearance of sulfate conjugate of morinidazole (substrate for OAT1 and OAT3) and the glucuronide conjugates (substrates for OAT3) [90]. Cadaveric kidneys from patients with postischemic acute renal failure sampled from allografts 1 h after reperfusion during transplant operation, revealed downregulation of OAT1 (evidenced by immunohistochemistry). Immunohistochemistry analysis showed reduction not only in the carrier protein levels but also its maldistribution, i.e., disappearance of lateral distribution, diffusion of cytoplasmic aggregates, presence of apical cytoplasmic aggregates, and disappearance of the staining in comparison with basolateral membrane staining of proximal tubule cells in the controls [91].

Dysfunction of BCRP (a major urate transporter, expressed also in the gastrointestinal tract) produced by genetic polymorphisms in the transporter regulatory gene was associated with elevated uric acid levels in human subjects. A trans-ancestry genome-wide association study demonstrated hepatocyte nuclear factor 1α (HNF1α) and HNF4α as potential transcriptional master regulators of urate levels. Experimental confirmation study revealed that HNF4α transactivated the promoter of *ABCG2* (BCRP) in kidney cells, and demonstrated that *HNF4A* Thr139Ile variant was functional [92].

Animal studies, using different models of kidney dysfunction/failure, e.g., 5/6 nephrectomized animals (model of chronic renal failure), ischemia/reperfusion-induced acute renal failure (model of acute renal failure), cisplatin- or glycerol-induced acute renal failure provide evidence that dysfunctional state of the organ is characterized by downregulation of uptake carriers (Oats) and increased function of efflux transporters (Mrp2, Mrp4, and P-gp) [89,93,94,95].

However, it is not clear whether the activity of renal transporters decreases in parallel with decreases in glomerular filtration or if any additional changes may occur. A recent analysis of clinical studies in patients with chronic kidney disease (CKD) involving 18 known substrates of OAT1 (*SLC22A6*) and OAT3 (*SLC22A8*) revealed that the active secretion of most drugs deteriorated faster than renal filtration in CKD, which meant that the secretory clearance normalized to GFR was reduced in CKD patients in comparison with subjects with normal renal function. In this study, however, function was analyzed, to which not only levels but also transporter activity contributes. Several uremic solutes (hippuric acid, phenylacetic acid, 3-carboxy-4-methyl-5-propyl-2-furanopropanoic acid-CMPF, and uric acid) were found to have the potential to inhibit OAT1 and OAT3 in humans (with hippuric acid being the most potent) [96]. A similar analysis of six OCT2 (*SLC22A2*) substrates revealed declined clearance, which paralleled GFR in CKD patients. The uremic solutes at concentrations exceeding the highest concentrations in CKD patients were able to inhibit the carrier function. Those findings may explain stable, not deteriorated, OCT2 function in patients with severe CKD (on the contrary to OAT1 and OAT3) [97].

## 7. Conclusions

Chronic kidney disease affects expression and function of drug carriers and transporters in the organ and thus reduces its elimination capacity of both endogenous (uremic solutes) and exogenous (drugs and toxins) substrates. The function of basolateral uptake carriers (OAT1 and OAT3) seems to precede dysfunction of efflux transporters and deteriorates faster than glomerular filtration in the course of chronic kidney disease. The renal carriers and transporters are also targets of drugs used in clinical practice, and intentional drug–drug interactions in the kidney are produced to increase therapeutic efficacy. The understanding of membrane carriers and transporters function in CKD is important not only to better characterize drug pharmacokinetics, drug actions, or drug–drug interactions in the kidney but also to define the organ pathophysiology.

## Figures and Tables

**Table 1:** Selected substrates of the main transporters and carriers presented in the review.

Transporter	Substrate	
	Endogenous	Drugs
ABC transporters		
*ABCB1*/MDR1	Aldosterone, β-amyloid, corticosterone, cortisol	Actinomycin D, amitriptyline, amprenavir, atorvastatin, carbamazepine, celiprolol, chlorpromazine, clopidogrel, citalopram, colchicine, cyclosporin A, daunorubicin, dexamethasone, digoxin, diltiazem, doxycycline, doxorubicin, erythromycin, etoposide, fexofenadine, imatinib, indinavir, irinotecan, itraconazole, ketoconazole, lamotrigine, lansoprazole, levetiracetam, levofloxacin, loperamide, losartan, lovastatin, melphalan, methylprednisolone, mitomycin C, mitoxantrone, morphine, nelfinavir, omeprazole, ondansetron, paclitaxel, pantoprazole, pentazocine, phenobarbital, phenothiazine, phenytoin, propranolol, quinidine, ranitidine, rhodamine-123, rifampicin, ritonavir, saquinavir, simvastatin, sirolimus, sparfloxacin, tacrolimus, talinolol, 99mTc-MIBI, teniposide, terfenadine, tetracycline, topotecan, vecuronium, verapamil, vinblastine, vincristine
P-glycoprotein		
*ABCC2*/MRP2	Bilirubin-G, estrone-3-S, glutathione, prostaglandin A2-GS	Adefovir, aflatoxin B1-epoxide-GS, ampicillin, azithromycin, ceftriaxone, cidofovir, cisplatin, cyclophosphamide-GS, dinitrophenyl-GS, doxorubicin, doxorubicin-GS, epirubicin, estradiol 17βD-G, etacrynic acid-GS, etoposide-G, etoposide, hydroxynonenal-GS, hyodeoxycholate-G, indinavir, irinotecan, lopinavir, melphalan-GS, methotrexate, mitoxantrone, nelfinavir, olmesartan, ritonavir, saquinavir, SN-38-G (irinotecan metabolite), valsartan, vinblastine, vincristine
*ABCC4*/MRP4	Bile salts, conjugated steroids, folate, glycocholate, taurocholate, urate	6-mercaptopurine, 6-thioguanine, acyclovir, adefovir, cefazolin, ceftizoxime, furosemide, hydrochlorothiazide, leucovorin, methotrexate, olmesartan, PAH, para-methoxy-N-ethylamphetamine, ritonavir, tenofovir, topotecan
*ABCG2*/BCRP	Urate	Canertinib, cimetidine, gefitinib, glyburide, imatinib, irinotecan, lamivudine, methotrexate, mitoxantrone, nilotinib, nitrofurantoin, pantoprazole, prazosin, rosuvastatin, sulfasalazine, topotecan
SLC carriers		
*SLC22A2*/OCT2	Acetylcholine, berberine, bile acids, choline, creatinine, dopamine, epinephrine, guanidine, histamine, norepinephrine, serotonin	Aflatoxin B1, amantadine, amiloride, cimetidine, cisplatin, D-tubocurarine, ethidium bromide, famotidine, ifosfamide, lamivudine, memantine, metformin, oxaliplatin, pancuronium, paraquat, pindolol, propranolol, ranitidine, varenicline, zalcitabine
*SLC22A4*/OCTN1	Acetylcholine, carnitine	Doxorubicin, entecavir, ergothioneine, gabapentin, imatinib, metformin, mitoxantrone, oxaliplatin, pregabalin, pyrilamine, quinidine, tiotropium, ipratropium, verapamil
*SLC22A5*/OCTN2	Carnitine	Cephaloridine, emetine, entecavir, etoposide, imatinib, ipratropium, spironolactone, tiotropium, verapamil
*SLC22A6*/OAT1	Cyclic nucleotides (cAMP, cGMP), folates, indoksyl sulfate, PGE2, PGF2α, uric acid	Adefovir, cephaloridin, ciprofloxacin, methotrexate, pravastatin, zidovudine
*SLC22A7*/OAT2	cGMP, creatinine, DHEAS, estrogen sulphate, PGE2, uric acid	5-fluorouracil, acyclovir, bumetadine, diclofenac, entecavir, ganciclovir, irinotecan, PAH, penciclovir, tetracycline, zidovudine
*SLC22A8*/OAT3	Uric acid, bile acids, conjugated hormones	Adefovir, cefaclor, ceftizoxime, cephaloridine, ciprofloxacin, conjugated sex steroids, methotrexate, NSAIDs, pravastatin, PGE2, zidovudine
*SLC47A1*/MATE1	Creatinine, estrone-S, guanidine, thiamine	Acyclovir, cephalexin, cephradine, cimetidine, fexofenadine, ganciclovir, metformin, MPP, paraquat, oxaliplatin, TEA, topotecan
SLC47A2/MATE2-K	Creatinine, carnitine, estrone-S, thiamine	Acyclovir, cimetidine, ganciclovir, lamivudine, metformin, oxaliplatin, quinidine, topotecan
*SLCO4A1*/OATP4A1	ADMA, cAMP, chenodeoxycholate, estrone-3-S, glycoycholate, T3, T4	Digoxin, ouabain, methotrexate

Sulfate conjugates (-S), glutathione conjugates (-GS), glucuronide conjugates (-G), 99mTc-MIBI—(99mTc) methoxyisobutylisonitrile, ADMA—asymmetrical dimethylarginine, cAMP—cyclic adenosine monophosphate, cGMP—cyclic guanosine monophosphate, DHEAS—dehydroepiandrosterone, MPP—1-methyl-4-phenylpyridinium, NSAID—nonsteroidal anti-inflammatory drug, PAH—paaaminohippurate, PGE2—prostaglandin E2, PGF2α—prostaglandin 2α, T3—triiodothyronine, T4—thyroxine, TEA—tetraethylammonium, SLC—solute carriers, ABC—ATP-binding cassette transporters.

## Data Availability

Not applicable.

## Abbreviations

ABC	ATP-binding cassette transporter
ADMA	asymmetric dimethylarginine
BCRP	breast cancer resistance protein
CrCl	creatinine clearance
CKD	chronic kidney disease
CMPF	3-carboxy-4-methyl-5-propyl-2-furanopropanoic acid
CRF	chronic renal failure
DDI	drug–drug interaction
ENT1/*SLC29A1*	equilibrative nucleoside transporter 1
ENT2/*SLC29A2*	equilibrative nucleoside transporter 2
FDA	US Food and Drug Administration
GFR	glomerular filtration rate
GLUT9/*SLC2A9*	facilitative glucose transporter 9
GSA	guanidine succinate
GSH	glutathione
HNF1α/*HNF1A*	hepatocyte nuclear factor 1α
HNF4α/*HNF4A*	hepatocyte nuclear factor 4α
MATE1/*SLC47A1*	multidrug and toxin extrusion protein 1
MATE2/*SLC47A2*	multidrug and toxin extrusion protein 2
MATE2-K/*SLC47A2*	multidrug and toxin extrusion protein 2 kidney-specific
MRP1/*ABCC1*	multidrug resistance-associated protein 2
MRP2/*ABCC2*	multidrug resistance-associated protein 2
MRP3/*ABCC3*	multidrug resistance-associated protein 3
MRP4/*ABCC4*	multidrug resistance-associated protein 4
MRP5/*ABCC5*	multidrug resistance-associated protein 5
MRP6/*ABCC6*	multidrug resistance-associated protein 6
MTX	methotrexate
NSAIDs	non-steroidal anti-inflammatory drugs
OAT1/*SLC22A6*	organic anion transporter 1
OAT2/*SLC22A7*	organic anion transporter 2
OAT3/*SLC22A8*	organic anion transporter 3
OAT4/*SLC22A11*	organic anion transporter 4
OATP1A2/*SLCO1A2*	kidney organic anion transporting polypeptide 1A2
OATP2B1/*SLCO2B1*	kidney organic anion transporting polypeptide 2B1
OATP4C1/*SLCO4C1*	kidney organic anion transporting polypeptide 4C1
OCT1/*SLC22A1*	organic cation transporter 1
OCT2/*SLC22A2*	organic cation transporter 2
OCT3/*SLC22A3*	organic cation transporter 3
OCTN1/*SLC22A4*	organic cation/carnitine transporter 1
OCTN2/*SLC22A5*	organic cation/carnitine transporter 2
OSTα/*SLC51A*	organic solute transporter α
OSTβ/*SLC51B*	organic solute transporter β
PAH	p-aminohippurate
PAN	puromycin aminonucleoside
PEPT1/*SLC15A1*	peptide transporter 1
PEPT2/*SLC15A2*	peptide transporter 2
P-gp. MDR1/*ABCB1*	glycoprotein, multidrug resistance protein 1
PMAT/*SLC29A4*	plasma membrane amino-acid transporter
SGLT2/*SLC5A2*	sodium-glucose co-transporter 2
SLC	solute carrier
SLCO4A1/*OATP4A1*	kidney organic anion transporting polypeptide 4A1
URAT1/*SLC22A12*	urate transporter 1
XO	xanthine oxidase
